# Seroprevalence of HCV, HBV and HIV in two inner-city London emergency departments

**DOI:** 10.1017/S0950268819000360

**Published:** 2019-03-12

**Authors:** L. Cieply, R. Simmons, S. Ijaz, E. Kara, A. Rodger, W. Rosenberg, A. McGuinness, J. L. Mbisa, J. Ledesma, N. Ohemeng-Kumi, S. Dicks, H. Potts, S. Lattimore, S. Mandal

**Affiliations:** 1 Immunisation, Hepatitis and Blood Safety Department, Centre for Infectious Disease Surveillance & Control (CIDSC), National Infection Service, Public Health England, London, UK; 2 The National Institute for Health Research Health Protection Research Unit (NIHR HPRU) in Blood Borne and Sexually Transmitted Infections at University College London, London, UK; 3 Blood Borne Virus Unit, Virus Reference Department, National Infection Service, Public Health England, London, UK; 4 The Royal Free London NHS Foundation Trust, The Royal Free Hospital, London, UK; 5 Institute for Global Health, University College London, London, UK; 6 University College London Hospitals NHS Foundation Trust, University College London Hospital, London, UK; 7 Antiviral Unit, Virus Reference Department, National Infection Service, Public Health England, London, UK; 8 Institute of Health Informatics, University College London, London, UK

**Keywords:** Anonymous testing, emergency department, hepatitis B, hepatitis C, HIV

## Abstract

Summary: In this paper we build on work investigating the feasibility of human immunodeficiency virus (HIV) testing in emergency departments (EDs), estimating the prevalence of hepatitis B, C and HIV infections among persons attending two inner-London EDs, identifying factors associated with testing positive in an ED. We also undertook molecular characterisation to look at the diversity of the viruses circulating in these individuals, and the presence of clinically significant mutations which impact on treatment and control.

Blood-borne virus (BBV) testing in non-traditional settings is feasible, with emergency departments (ED) potentially effective at reaching vulnerable and underserved populations. We investigated the feasibility of BBV testing within two inner-London EDs. Residual samples from biochemistry for adults (⩾18 years) attending The Royal Free London Hospital (RFLH) or the University College London Hospital (UCLH) ED between January and June 2015 were tested for human immunodeficiency virus (HIV)Ag/Ab, anti-hepatitis C (HCV) and HBsAg. PCR and sequence analysis were conducted on reactive samples. Sero-prevalence among persons attending RFH and UCLH with residual samples (1287 and 1546), respectively, were 1.1% and 1.0% for HBsAg, 1.6% and 2.3% for anti-HCV, 0.9% and 1.6% for HCV RNA, and 1.3% and 2.2% for HIV. For RFH, HBsAg positivity was more likely among persons of black *vs.* white ethnicity (odds ratio 9.08; 95% confidence interval 2.72–30), with anti-HCV positivity less likely among females (0.15, 95% CI 0.04–0.50). For UCLH, HBsAg positivity was more likely among non-white ethnicity (13.34, 95% CI 2.20–80.86 (Asian); 8.03, 95% CI 1.12–57.61 (black); and 8.11, 95% CI 1.13–58.18 (other/mixed)). Anti-HCV positivity was more likely among 36–55 year olds *vs.* ⩾56 years (7.69, 95% CI 2.24–26.41), and less likely among females (0.24, 95% CI 0.09–0.65). Persons positive for HIV-markers were more likely to be of black *vs.* white ethnicity (4.51, 95% CI 1.63–12.45), and less likely to have one ED attendance (0.39, 95% CI 0.17–0.88), or female (0.12, 95% CI 0.04–0.42). These results indicate that BBV-testing in EDs is feasible, providing a basis for further studies to explore provider and patient acceptability, referral into care and cost-effectiveness.

## Introduction

There are an estimated 180 000 persons with chronic hepatitis B (HBV) and 210 000 with chronic hepatitis C (HCV) in the UK, roughly equating to a prevalence of 0.4% for HBV and for HCV in 2018 [1, 2]. Similarly an estimated 101 600 persons were living with human immunodeficiency virus (HIV) in the UK in 2017, equivalent to an estimated prevalence of 0.2% [3]. The undiagnosed fraction for HIV for 2017 was estimated at 8%, however while unknown for HCV and HBV it is likely substantial. The annual unlinked anonymous survey in people who inject drugs (PWID) estimates that half of those injecting psychoactive drugs are unaware of their HCV-positive status (HCV report). The low diagnosed fraction for hepatitis is likely related to the fact that many of those at risk (e.g. PWID, recent migrants, prisoners, men who have sex with men) are socially marginalised and underserved and so less likely to engage with traditional primary care settings. Many barriers exist that contribute to underdiagnosis including social and individual barriers, such as lack of understanding of their risk, fear of the diagnosis and implications, concerns about social stigma attached to the infection and lack of knowledge about local healthcare [4, 5]; structural barriers such as lack of easily accessible and convenient community services and long waiting times for appointments; and system barriers created by complex commissioning and referral pathways as well as lack of health professional awareness [6–8].

Testing for HIV has traditionally been targeted in risk groups such as persons attending genitourinary medicine (GUM) services or as part of universal antenatal screening programme because of the risk of mother to child transmission. In the last decade, there has been a drive to reduce barriers to HIV testing in the UK through national recommendations to expand offer of HIV test to non-traditional settings, such as new registrations in general practice, all medical admissions and accident and emergency in areas where HIV diagnosed prevalence was estimated at >2 per 1000, encouraging potentially high-risk individuals to take up testing who would not otherwise have been reached [1, 9, 10], and aligning with NICE recommendations for universal testing where HIV prevalence is ⩾0.2% [11].

NICE guidance in 2012 on improving offer and uptake of testing for HBV and HCV includes recommended testing in primary care, prisons and immigration removal centres, drug services and sexual health and GUM clinics [12], with WHO recommending universal testing where HCV prevalence is ⩾5.0% for HCV and ⩾2.0% for HBV [13]. While the offer of a HBV and HCV test in primary care is recommended for migrants from medium or high prevalence countries (i.e. prevalence above 2%) [14], these recommendations are not being consistently implemented in GP practices due to lack of awareness, workload and resource issues [15]. Although EDs have not been explicitly mentioned in the NICE guidance for HBV and HCV, it is a setting which has been explored for HIV and TB testing, and so could be considered for HBV and HCV. The ED setting is universally accessible to all people [16–18], and therefore could be potentially effective at reaching more vulnerable and underserved populations [4].

A number of studies investigating blood-borne virus (BBV) testing in EDs have been carried out to look at testing implementation and uptake and prevalence of BBV in those tested. Many studies focused on HIV testing uptake ranged from 30% to 65%, with the proportion newly diagnosed ranging from 0.06% to 0.3% [19–22]. In 2014, nine UK EDs took part in a week long campaign entitled ‘Going Viral’, in which combined BBV tests were offered to patients attending the ED. During that week, 27% of eligible patients accepted testing, with 0.8%, 0.7% and 1.8% diagnosed HIV, HBV and HCV, respectively, with the corresponding proportion newly diagnosed 0.3%, 0.5% and 0.7%, respectively [23]. Finally, among attendees at St Thomas’ ED, London 2016, half of persons accepted a test for either HBV or HCV, with a positivity of 1.1% and 2.2%, respectively [24].

While the test yields in most studies are reasonably high – above background population rates, the variable offer and uptake of testing renders it difficult to make any direct inferences about prevalence of BBV in patients attending ED but does hint at implementation challenges such as patient and provider attitudes, entrenched practices – all of which need to be considered in an assessment of feasibility. Equally important as expanding testing to identify the undiagnosed population is linkage to care and treatment; this being the ultimate goal of BBV testing strategies to improve outcomes in the individual but also to realise public health impact of treatment as prevention. This is particularly relevant for HCV infection with the availability of direct acting antiviral (DAA) which can cure infection in most people [25].

Our study aims to estimate the prevalence of HBV, HCV and HIV infections among those persons attending two inner-city London EDs, identifying factors associated with testing positive in an ED and describing the molecular characteristics of those BBV-infected ED attendees. The Royal Free London NHS Foundation Trust and University College Hospital Foundation Trust serve very diverse populations, with a high number of attendances. Local populations served by these hospitals include Camden, Islington, Barnet, Enfield, Haringey and Westminster, of which three out of six have high prevalence estimates for HIV and HCV [26, 27], high rates of HCV infections among persons injecting drugs [27] and diverse deprivation scores [28, 29]. Undertaking molecular characterisation provides an opportunity to look at the diversity of the viruses circulating in these individuals and will allow for some comment to be made on the presence of clinically significant mutations which may impact on treatment and control.

## Methods

### Samples

Between January and June 2015, adults (⩾18 years) attending the ED at either The Royal Free London NHS Foundation Trust or the University College London NHS Foundation Trust, on whom blood samples were taken for biochemistry as part of their routine care, were included in the study. Residual sera/plasma of sufficient quantities were sent to the Blood-borne Virus Unit (BBVU) at Public Health England, Colindale for testing.

The study was undertaken as part of the National Institute for Health Research (NIHR) Health Protection Research Unit (HPRU) in Blood Borne and Sexually Transmitted Infections at University College London.

### Virological testing

Serological testing was undertaken on the Architect platform (Abbott, Maidenhead, UK) for the following markers: HIV 1 and 2 Ag/Ab, anti-HCV and HBsAg. The tests were performed in accordance to the manufacturers’ instructions. However, as the samples originated from biochemistry laboratories using automated liquid handlers, a decision was made to consider only samples yielding signals five times above the cut-off as being reactive to minimise any risks from cross-contamination.

Nucleic acid was extracted using QIAsymphony virus/pathogen DSP mini kit (Qiagen) for those samples with serological evidence of HIV and/or HCV infections and using MagNApure 96 DNA Viral Nucleic Acid Small Volume kit (Roche Diagnostics, Mannheim, Germany) for the samples with HBV markers. The viral load was quantified by real-time PCR as previously reported [30–32] and the extracts were analysed by PCRs for HIV [33], HBV and HCV genotyping [34]. Next-generation sequencing and a bioinformatic analysis were performed on the positive samples as described [35]. Consensus sequences were aligned with reference sequences for HIV, HCV and HBV using MEGA6 [36] and a phylogenetic analysis were done using the neighbour-joining distance matrix algorithm and the Kimura-2 parameters model. The drug susceptibility of HCV and HIV samples was determined using Geno2pheno[HCV] [37] and the Stanford HIV drug resistance database genotypic interpretation algorithm [38], respectively.

### Demographics

A minimum dataset of demographic data (age; sex; ethnicity; time, day and month of attendance; reason for attendance; and GP registration) was extracted from the trusts’ Patient Administrative Systems (PAS) and linked to the subsequent test results. Data on all ED attendees were collected to determine how representative the tested population was. Patient's demographics (age, sex and ethnicity) as well as time and reason for attendance were compared. Reason for attendance was reported using Systematized Nomenclature of Medicine-Clinical Terms (SNOMED) and was grouped into 14 reasons to mirror those used in a week long campaign entitled ‘Going Viral’, (in which combined BBV tests were offered to patients attending nine UK EDs) to allow for comparison [23]; trauma and orthopaedics, infections, cardiac conditions, haematology, central nervous system (CNS) conditions, respiratory conditions, gastrointestinal conditions, urological conditions, obstetrics and gynaecological conditions, diabetes, dermatology conditions and allergy, maxillofacial, ENT conditions and ophthalmology, psychological and social, and not reported. Persons were classified as re-attending if there was more than one attendance recorded during the study period.

### Pseudo-anonymisation and unlinking

Samples were pseudonymised and assigned a unique study ID. After linking with the PAS extract and prior to testing samples, all individual hospital and laboratory numbers were irreversibly removed so that the final dataset did not contain sufficient information to identify individuals. Only one sample per individual was included in the analysis.

## Statistical analysis

Descriptive statistics were given for all persons presenting at an ED, and for persons testing positive for a BBV. Data were deduplicated prior to anonymisation and through sequencing if positive to ensure that only one result per individual was included within the analysis, as re-attenders testing positive would lead to an overestimate of seroprevalence, or an underestimate among re-attenders testing negative. The *χ*^2^ or the Fisher's exact test was used to compare categorical data. Ninety-five per cent confidence intervals (CI) were estimated. Factors associated with testing positive for a BBV were assessed through univariate logistic regression analysis of the following variables: gender, age, ethnicity, re-attendance, time, day and reason of attendance.

Statistical analyses were performed using STATA version 13.

## Ethics

Ethical approval was obtained for the Unlinked Anonymous Testing (UAT) approach via the Integrated Research Approval System (IRAS) proportionate review service. Specific consent was not sought as individual patients could not be linked to their test results and were not contacted and informed of their serological status.

## Results

During the 6-month period January–June 2015, 75 910 persons attended two emergency departments (ED) within London, 30 052 persons attended The Royal Free Hospital (RFH), and 45 858 attended the University College London Hospital (UCLH). Residual blood samples referred for BBV testing at PHE were available on 2833 persons overall: 1287 and 1546 persons from RFH and UCLH, respectively.

Table 1 presents the distribution of the 75 910 persons attending the two ED within the 6-month period, for both hospitals the majority of persons reported being registered with a GP, and of white ethnicity. The median age of persons attending RFH was 42 years (IQR: 29–61) and for the UCLH 35 years (IQR: 26–52). The commonest reason (over a third) for attendance at both EDs was trauma and orthopaedics, followed by gastrointestinal complaints.

**Table 1.** Demographics of persons attending two emergency departments in London, Royal Free Hospital and University College London Hospital, between January and June 2015

	Royal Free Hospital				University College London Hospital			
	ED attendees		Residual blood sample taken for biochemistry		ED attendees		Residual blood sample taken for biochemistry	
	*n* = 30 052	%	*n* = 1287	%	*n* = 45 858	%	*n* = 1546	%
Sex								
Male	14 170	47.2	608	47.2	22 515	49.1	749	48.4
Female	15 881	52.8	679	52.8	23 341	50.9	797	51.6
Not reported	1				2			
Age group								
18–35	11 758	39.1	284	22.1	23 694	51.7	503	32.5
36–55	8916	29.7	353	27.4	12 776	27.9	451	29.2
⩾56	9378	31.2	650	50.5	9388	20.5	592	38.3
Ethnicity								
White	17 912	64.8	784	65.0	20 829	70.3	829	73.1
Asian	3820	13.8	180	14.9	2885	9.7	96	8.5
Black	2561	9.3	109	9.0	2868	9.7	105	9.3
Other/mixed	3370	12.2	134	11.1	3053	10.3	104	9.2
Not reported	2389		80		16 223		412	
Re-attendance								
Yes	5652	18.8	552	42.9	6866	15.0	577	37.3
No	24 400	81.2	735	57.1	38 992	85.0	969	62.7
Registered with a GP								
Yes	28 058	93.4	1244	96.7	35 720	77.9	1319	85.3
No	1994	6.6	43	3.3	10 138	22.1	227	14.7
Time of attendance								
00:00–07:59	3512	11.7	247	19.2	6026	13.1	120	7.8
08:00–15:59	14 249	47.4	526	40.9	21 732	47.4	978	63.3
16:00–23:59	12 291	40.9	514	39.9	18 100	39.5	448	29.0
Day								
Weekday	21 800	72.5	800	62.2	34 308	74.8	1250	80.9
Weekend	8252	27.5	487	37.8	11 550	25.2	296	19.1
Reason for attendance								
Trauma and orthopaedics	9512	36.4	210	19.4	16 256	43.6	238	21.0
Infections	2640	10.1	112	10.4	2639	7.1	113	10.0
Cardiac conditions	1850	7.1	141	13.0	1119	3.0	56	4.9
Haematology	102	0.4	10	0.9	78	0.2	7	0.6
Central nervous system conditions	1286	4.9	72	6.7	3141	8.4	178	15.7
Respiratory conditions	1340	5.1	79	7.3	2122	5.7	100	8.8
Gastrointestinal conditions	3348	12.8	202	18.7	3996	10.7	234	20.6
Urological conditions	1556	5.9	103	9.5	1570	4.2	67	5.9
Obstetrics and gynaecological conditions	849	3.2	39	3.6	1637	4.4	44	3.9
Diabetes	115	0.4	10	0.9	129	0.3	5	0.4
Dermatology conditions and Allergy	892	3.4	31	2.9	1039	2.8	19	1.7
Maxillofacial, ENT conditions and ophthalmology	1831	7.0	43	4.0	2574	6.9	47	4.1
Psychological and social	842	3.2	29	2.7	978	2.6	26	2.3
Not reported	3889		206		8580		412	

Proportions are calculated where information has been reported.

Although a low proportion of persons attending the EDs during the study time period had biochemistry residual samples available for BBV testing (3.4% at UCL and 4.3% RFH; 3.7% overall), the distribution of persons by sex and ethnicity for whom a blood sample was taken was similar to that for all persons attending the EDs, with just over 50% being female and over 65% being white (Table 1). The majority of ED attendees reported being registered with a GP regardless of whether a residual sample was available, although a higher proportion were GP-registered among RFH compared with UCLH ED attendees. Compared with all ED attendees, persons for whom a blood sample was taken at both EDs were older, and more likely to re-attend. While all patients were more likely to attend on a weekday and between the hours 00:00–07:59 am at both EDs, these proportions were higher among those for whom a residual blood sample was available at UCLH. After trauma and orthopaedics, the most common reason for attendance among those with a residual sample was cardiac and urological at RFH, and CNS and infections at UCLH (Table 1).

The sero-prevalence rates for samples from RFH and UCLH, respectively, were 1.1% (*n* = 14) and 1.0% (*n* = 15) for HBsAg; 1.6% (*n* = 21) and 2.3% (*n* = 35) for anti-HCV; 0.9% (*n* = 12) and 1.6% (*n* = 25) for HCV RNA; and 1.3% (*n* = 17) and 2.2% (*n* = 34) for anti-HIV and p24Ag (Table 2). The median age for those testing positive was 55 years (IQR: 35–62), 57 years (IQR: 49–70), 60 years (IQR: 51–72) and 55 years (IQR: 48–63) for HBsAg, anti-HCV, HCV RNA, and anti-HIV and p24Ag, respectively, for persons attending RFH and 52 years (IQR: 30–63), 45 years (IQR: 38.5–54), 43 years (IQR: 38–52) and 43 years (IQR: 32–55) for HBsAg, anti-HCV, HCV RNA, and anti-HIV and p24Ag, respectively, for those attending UCLH. Four persons attending UCLH were HIV/HCV coinfected, with no coinfections identified among those attending RFH. Persons tested whilst attending RFH and UCLH ED are characterised in Tables 3 and 4, respectively.

**Table 2.** Sero-prevalence of BBV in persons attending two emergency departments in London, Royal Free Hospital and University College London Hospital, between January and June 2015, for whom residual blood samples were available

	ED attendees	Blood sample taken for biochemistry		HBsAg-positive		Anti-HCV-positive		HCV PCR-positive		Anti-HIV- and p24Ag-positive	
		*n*	%	*n*	%	*n*	%	*n*	%	*n*	%
Royal Free Hospital	30 052	1287	4.3	14	1.1	21	1.6	12	0.9	17	1.3
University College London Hospital	45 858	1546	3.4	15	1.0	35	2.3	25	1.6	34	2.2

**Table 3.** Demographics and positivity rate of persons attending The Royal Free Hospital emergency department between January and June 2015 for whom a residual sample was available and tested for BBV

	Persons tested	HBsAg-positive		Anti-HCV-positive		HCV PCR-positive		Anti-HIV- and p24Ag-positive	
	*n* = 1287	*n* = 12	%	*n* = 21	%	*n* = 12	%	*n* = 15	%
Sex									
Male	608	6	1.0	18	3.0	10	1.6	9	1.5
Female	679	6	0.9	3	0.4	2	0.3	6	0.9
Age group									
18–35	284	4	1.4	0	0.0	0	0.0	4	1.4
36–55	353	4	1.1	9	2.5	5	1.4	4	1.1
⩾56	650	4	0.6	12	1.8	7	1.1	7	1.1
Ethnicity									
White	784	5	0.6	10	1.3	4	0.5	10	1.3
Asian	180	1	0.6	4	2.2	2	1.1	3	1.7
Black	109	6	5.5	3	2.8	3	2.8	1	0.9
Other/mixed	134	0	0.0	2	1.5	1	0.7	1	0.7
Not reported	80	0	0.0	2	2.5	2	2.5	0	0.0
Re-attendance									
Yes	552	6	1.1	13	2.4	7	1.3	3	0.5
No	735	6	0.8	8	1.1	5	0.7	12	1.6
Registered with a GP									
Yes	1244	12	1.0	20	1.6	12	1.0	14	1.1
No	43	0	0.0	1	2.3	0	0.0	1	2.3
Time of attendance									
00:00–07:59	247	5	2.0	3	1.2	1	0.4	1	0.4
08:00–15:59	526	2	0.4	11	2.1	6	1.1	7	1.3
16:00–23:59	514	5	1.0	7	1.4	5	1.0	7	1.4
Day of attendance									
Weekday	800	7	0.9	14	1.8	7	0.9	10	1.3
Weekend	487	5	1.0	7	1.4	5	1.0	5	1.0
Reason for attendance									
Trauma and orthopaedics	210	1	0.5	3	1.4	1	0.5	2	1.0
Infections	112	1	0.9	2	1.8	0	0.0	1	0.9
Cardiac conditions	141	1	0.7	6	4.3	6	4.3	0	0.0
Haematology	10	0	0.0	0	0.0	0	0.0	0	0.0
Central nervous system conditions	72	0	0.0	0	0.0	0	0.0	1	1.4
Respiratory conditions	79	0	0.0	2	2.5	1	1.3	4	5.1
Gastrointestinal conditions	202	5	2.5	1	0.5	0	0.0	0	0.0
Urological conditions	103	0	0.0	3	2.9	1	1.0	1	1.0
Obstetrics and gynaecological conditions	39	0	0.0	0	0.0	0	0.0	0	0.0
Diabetes	10	0	0.0	0	0.0	0	0.0	0	0.0
Dermatology conditions and allergy	31	1	3.2	0	0.0	0	0.0	0	0.0
Maxillofacial, ENT conditions and ophthalmology	43	2	4.7	0	0.0	0	0.0	0	0.0
Psychological and social	29	0	0.0	0	0.0	0	0.0	0	0.0
Not reported	206	1	0.5	4	1.9	3	1.5	6	2.9

**Table 4.** Demographics and positivity rate of persons attending the University College Hospital London emergency department between January and June 2015 for whom a residual sample was available and tested for BBV

	Persons tested	HBsAg-positive		Anti-HCV-positive		HCV PCR-positive		Anti-HIV- and p24Ag-positive	
	*n* = 1546	*n* = 12	%	*n* = 24	%	*n* = 15	%	*n* = 25	%
Sex									
Male	749	6	0.8	19	2.5	10	1.3	22	2.9
Female	797	6	0.8	5	0.6	5	0.6	3	0.4
Age group									
18–35	503	5	1.0	4	0.8	3	0.6	8	1.6
36–55	451	3	0.7	17	3.8	10	2.2	11	2.4
⩾56	592	5	0.8	3	0.5	2	0.3	6	1.0
Ethnicity									
White	829	2	0.2	18	2.2	11	1.3	11	1.3
Asian	96	3	3.1	0	0.0	0	0.0	1	1.0
Black	105	2	1.9	1	1.0	1	1.0	6	5.7
Other/mixed	104	2	1.9	1	1.0	1	1.0	4	3.8
Not reported	412	3	0.7	4	1.0	2	0.5	3	0.7
Re-attendance									
Yes	577	6	1.0	14	2.4	9	1.6	15	2.6
No	969	6	0.6	10	1.0	6	0.6	10	1.0
Registered with a GP									
Yes	1319	8	0.6	18	1.4	10	0.8	22	1.7
No	227	4	1.8	6	2.6	5	2.2	3	1.3
Time of attendance									
00:00–07:59	120	2	1.7	3	2.5	2	1.7	2	1.7
08:00–15:59	978	6	0.6	14	1.4	9	0.9	16	1.6
16:00–23:59	448	4	0.9	7	1.6	4	0.9	7	1.6
Day of attendance									
Weekday	1250	10	0.8	20	1.6	12	1.0	20	1.6
Weekend	296	2	0.7	4	1.4	3	1.0	5	1.7
Reason for attendance									
Trauma and orthopaedics	238	2	0.8	7	2.9	6	2.5	6	2.5
Infections	113	0	0.0	2	1.8	1	0.9	2	1.8
Cardiac conditions	56	1	1.8	0	0.0	0	0.0	0	0.0
Haematology	7	0	0.0	0	0.0	0	0.0	0	0.0
Central nervous system conditions	178	1	0.6	2	1.1	2	1.1	3	1.7
Respiratory conditions	100	1	1.0	4	4.0	3	3.0	1	1.0
Gastrointestinal conditions	234	2	0.9	3	1.3	1	0.4	1	0.4
Urological conditions	67	1	1.5	0	0.0	0	0.0	0	0.0
Obstetrics and gynaecological conditions	44	2	4.5	0	0.0	0	0.0	0	0.0
Diabetes	5	0	0.0	0	0.0	0	0.0	0	0.0
Dermatology conditions and allergy	19	0	0.0	0	0.0	0	0.0	2	10.5
Maxillofacial, ENT conditions and ophthalmology	47	0	0.0	1	2.1	0	0.0	2	4.3
Psychological and social	26	0	0.0	1	3.8	0	0.0	2	7.7
Not reported	412	2	0.5	4	1.0	2	0.5	6	1.5

Nine (64%) and 10 (67%) of the HBsAg-reactive samples from RFH and UCLH, respectively, were HBV DNA-positive. Collective sequence and phylogenetic analysis showed the presence of all the five major HBV genotypes reflecting the diversity of the HBV-infected population in the UK (genotype A: *n* = 8, B: *n* = 1, C: *n* = 3, D: *n* = 2, E: *n* = 5). Where the information was available, the HBV genotype was consistent with the ethnicity of the individual. Similar analysis undertaken on the 12 and 25 HCV PCR-positive samples from RFH and UCLH, respectively, indicated the 1a (*n* = 14) and 3a (*n* = 11) viruses to be the most common with a range of other genotypes also noted (genotype 1b: *n* = 1, 2a: *n* = 1, 2b: *n* = 4, 3b: *n* = 1, 3 h: *n* = 1, 4a: *n* = 3, 4v: *n* = 1). The majority of HIV-reactive samples, 86% for RFH and 74% for UCLH, were RNA-negative with genotype determination possible in three samples, two of which were HIV-1 subtype B and one CRF02_AG. Additional analysis showed the presence of motifs linked to antiviral resistance in one sample.

We identified factors associated with a positive result using a univariable model; numbers were too small to use a multivariate model. Among persons attending the RFH ED, attendance information was available for 12/14 persons diagnosed as HBsAg-positive, all those diagnosed with HCV [20], and 15/17 persons diagnosed positive for anti-HIV and p24Ag. Persons testing positive for HBsAg were more likely to be of black ethnicity when compared with persons of white ethnicity (odds ratio (OR) 9.08; 95% CI 2.72–30). Persons testing anti-HCV-positive were less likely to be female when compared with men (OR 0.15, 95% CI 0.04–0.50 and OR 0.18, 95% CI 0.04–0.81 for anti-HCV and HCV PCR, respectively). There were no factors associated with testing anti-HIV- and p24Ag-positive (Table 5).

**Table 5.** Factors associated with testing positive for a BBV among persons attending The Royal Free Hospital emergency department between January and June 2015

	HBsAg-positive			Anti-HCV-positive			HCV PCR-positive			Anti-HIV- and p24Ag-positive		
	OR	95% CI	*P* value	OR	95% CI	*P* value	OR	95% CI	*P* value	OR	95% CI	*P* value
Sex												
Male	1		0.848	1			1		0.009	1		0.319
Female	0.89	0.29–2.79		0.15	0.04–0.50	<0.001	0.18	0.04–0.81		0.59	0.21–1.68	
Age group												
18–35	2.31	0.57–9.29					*			1.31	0.38–4.52	
36–55	1.85	0.46–7.45		1.39	0.58–3.33		1.32	0.42–4.19		1.05	0.31–3.62	0.912
⩾56	1		0.461	1		0.464	1		0.641	1		
Ethnicity												
White	1		0.002	1		0.632	1		0.227	1		0.227
Asian	0.87	0.10–7.50		1.76	0.55–5.67		2.19	0.40–12.06		1.31	0.36–4.82	
Black	9.08	2.72–30.27		2.19	0.59–8.09		5.52	1.22–25.00		0.72	0.09–5.65	
Other/mixed	*			1.17	0.25–5.41		1.47	0.16–13.22		0.58	0.07–4.58	
Re-attendance												
Yes	1		0.619	1		0.078	1		0.281	1		0.06
No	0.75	0.24–2.33		0.46	0.19–1.11		0.53	0.17–1.69		3.04	0.85–10.82	
Registered with a GP												
Yes	*			1		0.731	*			1		0.523
No	*			1.46	0.19–11.11		*			2.09	0.27–16.28	
Time of attendance												
00:00–07:59	5.41	1.04–28.10		0.38	0.08–1.74		0.35	0.04–2.94		0.3	0.04–2.46	
08:00–15:59	1		0.099	1		0.349	1		0.553	1		0.379
16:00–23:59	2.57	0.50–13.33		0.65	0.25–1.68		0.85	0.26–2.81		1.02	0.36–2.94	
Day of attendance												
Weekday	1		0.785	1		0.665	1		0.785	1		0.715
Weekend	1.18	0.37–3.72		0.82	0.33–2.04		1.18	0.37–3.72		0.82	0.28–2.41	
Reason for attendance												
Trauma and orthopaedics	1		0.26	1		0.22	1		0.072	1		0.264
Infections	1.88	0.12–30.39		1.25	0.21–7.62		*			0.94	0.08–10.45	
Cardiac conditions	1.49	0.09–24.06		3.07	0.75–12.47		9.29	0.75–12.47		*		
Haematology	*			*			*			*		
Central nervous system conditions	*			*			*			1.46	0.13–16.40	
Respiratory conditions	*			1.79	0.29–10.93		2.68	0.17–43.36		5.55	1.00–30.91	
Gastrointestinal conditions	5.3	0.61–45.81		0.34	0.04–3.33		*			*		
Urological conditions	*			2.07	0.41–10.44		2.05	0.13–33.09		1.02	0.09–11.38	
Obstetrics and gynaecological conditions	*			*			*			*		
Diabetes	*			*			*			*		
Dermatology conditions and allergy	6.97	0.42–114.35		*			*			*		
Maxillofacial, ENT conditions, and ophthalmology	10.2	0.90–115.08		*			*			*		
Psychological and social	*			*			*			*		

Among persons attending UCLH, attendance information was available for 12/15 persons diagnosed as HBsAg-positive, 24/35 persons diagnosed anti-HCV-positive, 15/25 of those diagnosed positive for HCV PCR and 25/34 persons diagnosed positive for anti-HIV and p24Ag. Persons testing positive for HBsAg were more likely to be of non-white ethnicity when compared with white ethnicity (OR 13.34, 95% CI 2.20–80.86; OR 8.03, 95% CI 1.12–57.61 and OR 8.11, 95% CI 1.13–58.18, respectively, for Asian, black and other/mixed ethnicity), persons testing positive for anti-HCV were less likely to be female when compared with males (OR 0.24, 95% CI 0.09–0.65), but more likely to be aged between 36 and 55 years for both persons anti-HCV-positive and PCR-positive when compared with 56 years and over (OR 7.69, 95% CI 2.24–26.41 and OR 6.69, 95% CI 1.46–30.68, respectively). Persons anti-HIV- and p24Ag-positive were also less likely to be female when compared with males (OR 0.12, 95% CI 0.04–0.42), were more likely to be of black ethnicity when compared with white ethnicity (OR 4.51, 95% CI 1.63–12.45) and less likely to have attended the ED only once during the 6-month period compared with multiple attendances (OR 0.39, 95% CI 0.17–0.88) (Table 6).

**Table 6.** Factors associated with testing positive for a BBV among persons attending the University College London Hospital emergency department between January and June 2015

	HBsAg-positive			Anti-HCV-positive			HCV PCR-positive			Anti-HIV- and p24Ag-positive		
	OR	95% CI	*P* value	OR	95% CI	*P* value	OR	95% CI	*P* value	OR	95% CI	*P* value
Sex												
Male	1		0.914	1		0.002	1		0.153	1		<0.001
Female	0.94	0.30–2.93		0.24	0.09–0.65		0.47	0.16–1.37		0.12	0.04–0.42	
Age group												
18–35	0.94	0.25–3.52		1.57	0.35–7.07		1.77	0.29–10.63		1.58	0.54–4.58	
36–55	0.79	0.19–3.31		7.69	2.24–26.41		6.69	1.46–30.68		2.44	0.90–6.65	
⩾56	1		0.945	1		<0.001	1		0.009	1		0.2
Ethnicity												
White	1		0.014	1		0.466	1		0.906	1		0.028
Asian	13.34	2.20–80.86		*			*			0.79	0.10–6.13	
Black	8.03	1.12–57.61		0.43	0.06–3.28		0.72	0.09–5.59		4.51	1.63–12.45	
Other/mixed	8.11	1.13–58.18		0.44	0.06–3.31		0.72	0.09–5.65		2.97	0.93–9.52	
Re-attendance												
Yes	1		0.37	1		0.036	1		0.074	1		0.021
No	0.59	0.19–1.85		0.42	0.19–0.95		0.39	0.14–1.11		0.39	0.17–0.88	
Registered with a GP												
Yes	1		0.104	1		0.182	1		0.069	1		0.695
No	2.94	0.88–9.84		1.96	0.77–5.00		2.95	1.00–8.71		0.79	0.23–2.66	
Time of attendance												
00:00–07:59	2.75	0.55–13.76		1.77	0.50–6.23		1.82	0.39–8.55		1.02	0.23–4.49	
08:00–15:59	1		0.505	1		0.708	1		0.758	1		0.994
16:00–23:59	1.46	0.41–5.20		1.09	0.44–2.73		0.97	0.30–3.17		0.95	0.39–2.34	
Day of attendance												
Weekday	1			1		0.752	1		0.933	1		0.913
Weekend	0.84	0.18–3.87		0.84	0.29–2.48		1.06	0.30–3.77		1.06	0.39–2.84	
Reason for attendance												
Trauma and orthopaedics	1		0.694	1		0.628	1		0.228	1		0.089
Infections	*			0.59	0.12–2.91		0.35	0.04–2.90		0.7	0.14–3.51	
Cardiac conditions	2.15	0.19–24.09		*			*			*		
Haematology	*			*			*			*		
Central nervous system conditions	0.67	0.06–7.41		0.38	0.08–1.83		0.44	0.09–2.20		0.66	0.16–2.69	
Respiratory conditions	1.19	0.11–13.30		1.38	0.39–4.81		1.2	0.29–4.88		0.39	0.05–3.29	
Gastrointestinal conditions	1.02	0.14–7.28		0.43	0.11–1.68		0.17	0.02–1.39		0.17	0.20–1.39	
Urological conditions	1.79	0.16–20.02		*			*			*		
Obstetrics and gynaecological conditions	5.62	0.77–40.99		*			*			*		
Diabetes	*			*			*			*		
Dermatology conditions and allergy	*			*			*			4.55	0.85–24.27	
Maxillofacial, ENT conditions and ophthalmology	*			0.72	0.09–5.97		*			1.72	0.34–8.79	
Psychological and social	*			1.32	0.16–11.17		*			3.22	0.62–16.85	

## Discussion

To our knowledge, this is the first study to estimate seroprevalence as well as describe the viral diversity of all three BBVs ED attendees through untargeted residual blood sample testing in two central London EDs. While not an assessment of feasibility of BBV testing in itself, our findings contribute to the growing body of evidence assessing how feasible, acceptable and economically viable BBV testing is within the ED setting. During the 6-month study period, the seroprevalence rates of BBVs among persons attending two EDs in London, who had blood taken as part of routine care and a residual sample available for testing, were 1.1% and 1.0% for HBsAg, 1.6% and 2.3% for anti-HCV, 0.9% and 1.6% for HCV RNA and 1.3% and 2.2% for anti-HIV and p24Ag, within the RFH and UCLH EDs, respectively. These proportions are higher than general population prevalence estimates and so provide some support of the assumption that higher risk groups for BBV may attend urban EDs. Seroprevalences in our study are not necessarily directly comparable with other studies estimating seropositivity in EDs as many of these studies actually measure positivity rates through targeted or risk-based testing and so do not actually estimate seroprevalence among all ED attendees. Additionally, the impact of new curative treatments, e.g. DAAs for HCV and ramp up of case finding may result in lower seroprevalences in the ED-attending population.

Albeit undertaken on limited numbers, molecular characterisation demonstrated the diversity of viruses circulating in BBV-infected populations, noting that genotypic information is relevant for the choice of antiviral treatment in many of these patients. The expected availability of HCV pan-genotypic therapies will remove this limitation to treatment, but there is a need for vigilance for anti-viral resistance motifs in circulating strains.

Due to patients attending ED not all requiring biochemistry tests done, compounded by laboratory work load and changes in staff which impacted on implementation of the study protocol in the laboratories, the proportion of biochemistry residual samples available for BBV testing among ED attendees was low. However, we actually were able to test almost 3000 samples, which is not an insignificant sample size. Reassuringly, the ED attendees from whom residual blood samples were obtained had overall similar characteristics (age, ethnicity and registration with GP, predominant reason for attendance) to the total ED-attending population in those hospitals indicating that they were generally representative of the ED population. A notable exception was that the attendees with blood samples were older and were more likely to re-attend. This likely corresponds to the fact that older persons are more likely to have health conditions that result in more acute hospital attendances and therefore have an episode of care in which blood tests are taken. Persons were de-duplicated to ensure only one attendance was presented within the results and represented persons not attendances.

Both hospitals within which the EDs reside are within the borough of Camden, London, which has high estimated prevalence for both HCV and HIV (152 and 38 per 100 000, respectively) [26, 27], alongside high rates of hospital admissions for HCV-related ESLD and HCC (13.3 per 100 000) [27]. The University of College London Hospital is also the main hospital for the borough of Islington, where prevalence for HCV is 75 per 100 000, and for HIV 32 per 100 000 [26, 27]. The borough of Islington contains a higher proportion of disadvantaged persons, with Islington ranked the 13th most deprived area in England compared with Camden which is ranked the 69th most deprived area [29]. Forty-four per cent of persons within the borough of Islington were living in the 20% most deprived areas, compared with 27% of those living in borough of Camden [28]. The borough of Camden is also situated next to Westminster, which has the highest number of rough sleepers in England [39], in whom increased risk of some BBV has been described [40]. The two areas of Camden have an average index of multiple deprivation, with average or higher levels for income, employment, education and crime. However, The Royal Free resides in an area with a health deprivation within the lowest 30% in England, and UCLH resides within an area that is in the lowest 20% for housing, and lowest 10% for living environment [29]. Those attending the ED for The Royal Free attendees were older than those attending UCLH, whereas UCLH attendees had a higher proportion of persons not registered with a general practitioner

The high proportion of persons anti-HIV-reactive, but HIV RNA-negative is suggestive of viral suppression because of antiretroviral treatment and implies that many of the people testing positive for HIV were already aware of their diagnosis and receiving treatment. However, although the UAT methodology meant it was not possible to differentiate persons previously undiagnosed, these findings were similar to the estimates, identified through opt-out testing at nine UK EDs across the UK where persons previously undiagnosed could be identified [23]. Ethnicity was identified as a factor associated with testing positive for HBsAg and HIV, with persons of black or Asian ethnicity more likely to test positive for HBsAg and black or other/mixed more likely to test positive for HIV. Unfortunately, country of birth could not be explored as it was poorly recorded on hospital systems. Sex and age were associated with testing positive for HCV, with males and persons aged 36–55 years being more likely to be infected.

Overall males were more likely to test positive for HCV and HIV which is consistent with published literature [17, 41–47] where males are identified at greater risk of BBVs through particular adult behavioural risk factors including sex between men, injecting drug use and homelessness (likely confounded by substance abuse and other risk factors). A higher proportion of HBV-infected persons were of non-white ethnicity compared with those testing positive for HCV and HIV, which, assuming some associations between country of birth and ethnicity in people attending EDs, is consistent with the known burden of chronic HBV among non-UK-born persons where acquisition was in high endemic countries prior to migration to the UK [48].

The median age of persons testing positive for all three BBV was older than the ED attendees, and in particular persons aged 36–55 years were disproportionally affected by HCV. This is consistent with laboratory reports for England and Wales where 53% of persons positive for anti-HCV were aged 35–54 years. Going Viral, a campaign to offer opt-out BBV testing to all adult patients who were already having a blood test as part of routine ED care, also found peak prevalence of HCV among men aged 35–44 years [23]. The authors go on to discuss targeted HCV testing for particular age groups, which has been recommended within the USA following high prevalence of undiagnosed HCV infections among persons born between 1945 and 1965.

Although this study suggests it is possible to identify people with BBVs within EDs, there are several aspects of operationalising testing in EDs that should be considered, including intrinsic (patient-specific) and extrinsic (structural, system and health worker) enablers and barriers to testing. Among staff, ‘lack of time’, ‘not a priority’ and ‘not medically indicated’ were reasons for not considering a patient for a test [49], and among registrars in a UK teaching hospital undertaking a survey for HIV testing practices, 40% indicated that they had not performed an HIV test on a patient they considered high risk, with the main contributing factor reported as uncertainties with regards counselling the patient [50]. Alternatively, a patient's perception of risk can also be a factor, when ED patients were asked about their perceived risk, only 37.9% of persons reporting high-risk behaviour recognised the need for an HIV test [51].

Although the ED is a setting which may identify those who would not ordinarily consider themselves at risk and therefore seek testing, linkage to a holistic range of health and social care services is a key component which cannot be ignored if better health outcomes are to be realised. It is important to ensure those diagnosed have access to BBV treatments which offer both individual and population benefits in terms of preventing disease progression (and for HCV cure) as well as preventing onward transmission. Equally vital is patient access to services supporting behavioural change and key preventative measures such as offering hepatitis A and B vaccination, provision of needle exchange and drug addiction treatment, and pre-exposure prophylaxis against HIV, as appropriate to the patient's needs.

Estimates of HCV cascade of care using surveillance data from sentinel English laboratories in 2005–2014 showed a low uptake of HCV treatment overall regardless of where the patient was tested (11.9%), with treatment uptake among those initially diagnosed in EDs 7.9%, higher than drug services and prisons where HCV testing is traditionally conducted (6.6% and 5.9%, respectively) [52]. Most BBV testing studies in EDs including an assessment of linkage to care were HIV-focused and conducted in the USA, with the proportion of individuals linked into care ranging from 29% to 100%, with individuals being lost before confirmatory testing if point of care tests were used, not returning for confirmatory results, leaving before preliminary results can be given, being uncontactable, or refusing treatment [23, 53–63]. Where HBV and HCV testing in EDs was the focus, linkage to care was worse for those with HCV as illustrated by the ‘VirA&Emic’ ED testing study at St Thomas’ Hospital in London, which found contacting patients following a diagnosis to ensure onward access to care difficult with two out of three of those HCV-positive either homeless or not registered with the correct address, resulting in only 33% of persons being contacted, compared with 52% for persons diagnosed with HBV [64]. Furthermore, of those who required further care, only one person diagnosed with HBV did not attend their appointment, compared with 11 of those diagnosed with HCV. The disparity between infections was also found in an opt-out ED screening programme in Dublin between 2014 and 2015, where linkage to care for new patients was higher for those diagnosed with HIV and HBV compared with HCV (100%, 95% and 74%, respectively) [63]. Finally, Orkin *et al*. [23] reported linkage to care for those newly *vs.* previously diagnosed, with 66% of those newly diagnosed linked into care, and 59% retained in care, whereas the respective figures for those previously diagnosed were 50% and 20%.

Although the UAT approach has its strengths, as it is more accurate and representative by eliminating selection bias by perceived risk or the way the test is offered, the UAT screening approach limits the ability to establish whether seropositive individuals had been previously diagnosed; this makes it difficult to demonstrate the ‘added value’ of BBV testing within this setting. However, the infection rates at these two EDs were similar to that of other ED studies where testing was actively offered, and higher than background population prevalence. Prevalence of BBV may, however, be underestimated in our study as not all attendances had blood taken as part of routine care. In addition, the findings of our study may not be generalisable to all EDs, in particular those situated outside of central London as the London boroughs for which these EDs are situated and serve have high HCV and HIV prevalence. Compared with other studies, a lower proportion of persons had biochemistry samples available which was likely due to not all ED patients having blood samples done, laboratory work load and changes in staff, so that not all biochemistry samples were aliquoted and sent to PHE. However, this would have occurred randomly rather than systematically.

In summary, this study's findings demonstrate that universal screening of ED attendees for all BBVs will yield positive cases in urban, diverse populations with relatively high prevalence. However, there is the need for further evaluation of the additional yield in terms of new diagnoses as opposed to repeat diagnoses, whether the population profiles of those tested within the ED differ from traditional settings, and whether disease prevalence thresholds for implementing BBV testing in an ED setting might be considered. High positivity rates and high utilisation of acute ED services among populations with particular demographics may equate to identification of vulnerable groups who do not seek healthcare in primary care and may also be at greater risk, or who may be unaware of their infection or have previously been diagnosed but have not re-engaged in care. Our findings lay the foundation for further studies to explore provider and patient acceptability, undiagnosed yield, linkage to care and cost-benefits of BBV testing in EDs, to enable targeted public health action to address the challenge of undiagnosed infections in vulnerable populations.
